# The Mediation Role of Self-Control in the Association of Self-Efficacy and Physical Activity in College Students

**DOI:** 10.3390/ijerph191912152

**Published:** 2022-09-26

**Authors:** Hongyan Yu, Li Yang, Jianing Tian, Larry Austin, Yiming Tao

**Affiliations:** 1 Department of Physical Education, Shanghai Jiao Tong University, Shanghai 200240, China; 2 Yunnan Province Sports Bureau, Kunming 650000, China; 3 International Division, Shanghai Gezhi Middle School, Shanghai 200240, China

**Keywords:** self-control, intention-behavior gap, physical activity, self-efficacy, college students, self-discipline, theory of planned behavior

## Abstract

Global COVID-19 lockdown measures have led to an apparent decrease in physical activity. This study aimed to explore the explanatory function of self-control’s mediating role between self-efficacy and physical activity among college students. The analysis used the data of 1627 university students (aged 19.41 ± 0.66, range 17–28, 40.5% males) at Shanghai Jiao Tong University. Self-efficacy, self-control, and physical activity were tested, respectively, by the general self-efficacy scale, the new brief self-control scale, and the International Physical Activity Questionnaire (IPAQ) scale, which were analyzed by SPSS software. Correlation analysis showed that self-efficacy, self-control, and physical activity were related in pairs. Comparing the two dimensions of self-control, we found that self-discipline mediated the relationship between self-efficacy and PA, and impulse control did not mediate the relationship. Regarding the gender difference according to multi-group analysis, findings showed that females need higher self-discipline from the path of self-efficacy to physical activity improvement than males.

## 1. Introduction

Physical activity (PA), an essential factor in sports, has consistently been associated with high psycho-social health in adolescents [1]. Studies indicate that exercise and physical activity are associated with better quality of life and mental health outcomes [2,3]. Irrefutable evidence has shown the effectiveness of PA in the primary and secondary prevention of several chronic diseases (e.g., cardiovascular disease, diabetes, cancer, hypertension, obesity, depression, and osteoporosis) and premature death [4]. A systematic review identified >850 articles and found strong evidence to support that PA could reduce adiposity, improve musculoskeletal and cardiovascular health, enhance fitness, and increase mental health for children and youth [5]. As a standard or parameter for adequate levels of PA, the World Health Organization (WHO) recommends that adults practice moderate PA for a minimum of 150 min per week, vigorous PA for 75 min per week, or a combination of the two [6]; however, one-third of adults and four-fifths of adolescents do not reach public health guidelines for the recommended levels of PA [7]. The decline of PA during adolescence was a consistent finding in the literature [8]. In particular, the COVID-19 restriction measures adopted worldwide have led directly to a decline in PA [9,10]. Thus, exploring the mechanism of improving PA among adolescents is necessary.

Self-efficacy (SE) is a judgment of the individual’s power to organize the actions necessary to perform a specific task [11]. Bandura proposed that the persistence and effort of individuals towards specific behaviors are closely related to their level of self-efficacy [12]. A stable positive correlation between self-efficacy and PA has been demonstrated [13,14]. It can also positively predict PA; for example, a study on the relationships between self-efficacy, PA, and health showed a significant positive impact among the three variables, which were mutually supported. The three variables could predict each other [15]. Another prospective study conducted among college students found that self-efficacy can directly predict PA, regardless of self-regulation [16]. The theory of planned behavior (TPB) is to explain how people achieve or change their behavior through thoughtful planning; it has been used successfully to predict and demonstrate a wide range of health behaviors and intentions, such as drinking, sleeping, and smoking, among others [17,18]. PA, especially habitual physical activity, is planned behavior that targets healthy living. Therefore, TPB can explain the SE to PA process.

In reality, individuals are often motivated to be physically active but have difficulties carrying out the behavior [19,20]; the intention–behavior gap is the gap between behavior intentions and behavior [21]. Hale, Householder, and Greene (2001) found a correlation of r = 0.39 to 0.45 between behavioral intention and actual behavior [22]. A study proves that an integrated model with TPB and facet-level models revealed that only 46% of the variance in exercise behavior and 70% in intention were explained [23]. Zhang insisted that cognitive variables such as attitudes, subjective norms, and a sense of personal control in the TPB explain the process of behavioral intention but fail to explain how these cognitive variables contribute to the occurrence and maintenance of behavior [24], yet this is the most critical aspect of facilitating physical activity.

Scholars believe that higher levels of self-control can fill the intention–behavior gap [25]. Self-control is a person’s capacity to control impulses and achieve higher-order goals through self-discipline [26]. A prospective study showed that higher traits of self-control implied a smaller intention-behavior gap [19]. Furthermore, some studies indicated that self-control plays a mediating role in exploring the “intention-behavior gap”. For example, Finne et al. (2019) followed a 13-week college physical education program. They concluded self-control moderated the intention-behavior gap and the association between willing and actual participants each week [27]. Self-control could be explained in the TPB that facilitates the onset of behavior. Still, it is unclear as to whether it can play a role in the relationship between self-efficacy and PA.

### 1.1. Self-Efficacy and Physical Activity

Some studies have indicated that SE correlates with PA. The correlation coefficient between SE and PA was 0.3 shown in a study by Casey et al. (2018) [28]. A latent variable structural equation modeling indicates that SE exhibited a cross-sectional relationship with PA among adolescent girls [29]. Ray (2010) argued that SE and self-reported PA positively correlate in children with congenital heart disease [30]. Dobbins et al. (2010) found that it was important for girls to promote continued PA by improving self-efficacy in combination with external validation and support [31].

Meanwhile, several scholars have illustrated that SE has long been a predictor of PA adoption and maintenance among healthy adults [16,32,33]. For example, Micky et al. (2017) proved that general self-efficacy fully mediates perceived barriers to physical activity and moderate- to high-intensity physical activity among 19- to 24-year-old health science students [34]. A systematic review reported that increasing SE was an effective method for increasing PA [35], and another review described how self-efficacy determines the consequences of PA and enhances exercise participation [36].

Azjen (1985) proposed the theory of planned behavior (TPB), rooted in the Theory of Reasoned Action (TRA), in 1980, to link an individual’s behavioral intention and actual behavior [37]. TPB differs from TRA by adding perceived behavioral control (PBC) to TRA; self-efficacy theory derived the concept [37]. PBC refers to an individual’s perception of the ease or difficulty of performing the behavior of interest [37]; it contains two components: the perceived ease of the behavior (self-efficacy) and the degree of one’s mastery of the behavior (sense of control) [38,39]. As one of the constructs of TPB, SE presents one’s ability to perform the behavior successfully; when people feel they can perform certain behaviors successfully, they are more likely to intend to do so. Thus, TPB has provided an excellent theoretical framework for explaining SE to improve PA.

### 1.2. Self-Control as a Mediating Mechanism

In TPB, behavioral intention is the key component, and components of attitude decide the power of behavioral intention and subjective and perceived behavioral control, which comprised together represent a person’s actual control over the behavior. Thus, behavioral compliment depends on both intention and behavioral control ability. According to the TPB model, increasing a person’s real behavioral control over behavioral intent or direct behavior can enhance behavior attainment. Still, the TPB does not elaborate on the precise control of behavior [38], which is why TPB is hard to explain the intention-behavior gap. Meanwhile, TPB states that internal and external factors influence all behaviors. PBC is the individual’s perceived control and the degree of difficulty in completing the behavior based on these factors (perceived easy/difficult), which determine whether or not to perform the behavioral goal. This process reflects the “process of behavioral selection”. This process provides a space for self-control. However, when faced with a choice, the instinctive, intuitive first response is the habitual response, often to satisfy people’s short-term desires. Self-control is the ability of people to restrain impulses, desires, and habitual reactions [40]; when short-term and long-term goals conflict, individuals restrain their ability to pursue long-term outcomes. For example, the ability to overcome laziness and keep exercising for a long time to be physically fit is self-control. Thus, the process of behavioral selection provides a space for self-control.

Self-control is one of the most widely studied concepts in psychological science [41], and it is associated with exercise and health-related behaviors [26,42]. Tangney et al. developed a brief self-control scale to assess a person’s self-control on the behavioral aspect in two subscales: self-discipline and impulse control. Adding self-control to the TPB model means increasing a person’s actual behavioral control over behavioral intentions to bridge the intention–behavior gap. Some studies have illustrated that self-control is a mediating mechanism influencing social behavior. For example, self-control in college students plays a partial and parallel mediating role between shyness and cell phone addiction [43]. Low self-control and aggression mediate adolescent inattention and Internet gaming disorder [44].

In addition, there are now studies that point to gender differences in all three variables of this model (self-efficacy, PA, and self-control). A study conducted in South Korea found persistent gender differences in adolescents’ levels of self-control, with males having lower self-control than females [45]. Secondly, an experiment conducted in 2022 with Chinese undergraduate students showed gender differences in self-efficacy, showing that the overall self-efficacy of female college students is lower than that of male college students [46]. Finally, studies by Cla and Sallis validated gender differences in PA [47,48].

### 1.3. The Present Study

On the basis of past research, studies have indicated that SE correlates with PA. The added construct of SE in TPB can provide theoretical support for their relationship, but the mechanism of self-control as a mediator between SE and PA is still unclear. In particular, the intention-behavior gap sometimes makes the facilitation of SE to PA unreliable. Several studies have confirmed that self-control can play a mediating role in the model; however, whether it can act on the relationship between SE and PA needs to be further investigated. Therefore, this study aimed to examine the association of self-efficacy and PA with college students and explore the mediating effect of self-control.

Understanding the relationship between self-control, self-efficacy, and PA would be beneficial in helping college students improve their physical and psychological health. We know that perceived behavioral control is not a person’s actual control over behavior; it does not explain how it controls and maintains behavior. It is one of the limitations presented in TPB [24,45]. Our study establishes the relationship between self-control and SE and PA, hopefully providing insight into understanding this issue.

Previous literature analysis proposes the following assumptions. In the study, the two dimensions of self-control, self-discipline, and impulse control are put into the model for analysis. Then, it compares the role of self-discipline and impulse control in self-efficacy and PA among university students. On the basis of previous literature analysis, we propose the following assumptions:

**Hypothesis** **1**: *Self-control (self-discipline and impulse control) mediated the relationship between self-efficacy and physical activity.*

**Hypothesis** **2**: *Gender moderates the effect of self-efficacy on physical activity through self-control.*

## 2. Materials and Methods

### 2.1. Participants and Procedures

Random sampling selected participants from Shanghai Jiao Tong University (SJTU) in China. After obtaining approval from the academic ethics committee of the first author’s university, we began the study. Questionnaire Star, a professional online platform, was used for data collection and obtaining the informed consent of each participant. We made a QR code linked to the questionnaire. The staff surveyed the national student fitness test of SJTU. Students were randomly scheduled from the registration system to come to the test. We recruited participants from the students who came to the test. The duration was from March to May 2021. We conducted the survey at the Shanghai Jiao Tong University Physical Fitness Testing Center. Participants randomly came and scanned the code on their cell phones, and informed consent for this survey appeared on the cell phone screen, informing them of the purpose, method, possible risks, benefits, data use, etc. The instructions remained on the cell phone screen for at least 10 s to ensure that participants had sufficient time to completely read and familiarize themselves with the experimental procedures. Then, participants who freely chose to “agree” were guided by the system to proceed with filling out the online questionnaire. Participants who chose to “disagree” were taken out of the process. Participants could opt out at any time throughout the process without any reason. The study protocol followed the guidelines of the Declaration of Helsinki and was approved by the Ethics Review Committee for Human Science and Technology of Shanghai Jiao Tong University, no. E2021100I. Participants volunteered for the study without recompense.

The RAOSOFT online calculator calculated the sufficient statistical power of the sample size [49]. On the basis of 17071 college students [50], a 3% margin of error, a 95% confidence level, and a 50% response distribution, the sample size should not be less than 1005. During the study period, the total sample at baseline was 5712 students, and 2310 students responded and completed the survey. After raw data cleaning (same answer in a row or regularity or incomplete in answering), 1627 valid data points remained and were entered into the analysis. All the subjects were students at university, with 968 males accounting for 59.5% and 659 females accounting for 40.5%. All participants’ mean age was 19.41 years (SD = 0.66, range 17–28).

### 2.2. Measures

#### 2.2.1. Self-Efficacy Scale

We used the general self-efficacy scale (GSES) to measure the participants’ self-efficacy, adapted by Zhang and Schwarzer from the English version [51]. The scale consisted of 10 affirmative items describing an individual’s general belief of success in coping with particularly difficult problems or novel situations, such as “I will be able to achieve most of the goals that I have set for myself” and “When facing difficult tasks, I am certain that I will accomplish them”. We provided a 4-point Likert scale to each item, ranging from 1 (not true at all) to 4 (exactly true). The higher the mean value of the 10 items, the higher the self-efficacy level. Previous research has shown the favorable reliability and validity of the general self-efficacy scale among Chinese adolescents [52] and other Chinese groups [53,54,55]. In this study, Cronbach’s alpha on the whole scale was 0.877.

#### 2.2.2. Brief Self-Control Scale

This study used the 7-item new brief self-control scale (BSCS) as a measurement of self-control ability [56]. The scale consisted of two dimensions of self-control that measured self-discipline (e.g., “I am good at resisting temptation”) and impulse-control (e.g., “I do certain things that are bad for me if they are fun”), each with three items and four items. We used a 5-point Likert scale ranging from 1 (not like me at all) to 5 (very much like me). We calculated the average score of the seven items: the higher the score, the higher the level of self-control. The sub-dimension scores were obtained by calculating the total scores of the items corresponding to each sub-dimension. The Chinese version, translated and revised by Luo Tao et al., has a good reliability with an internal consistency coefficient Cronbach’s alpha of 0.83 and alpha coefficients of 0.85 and 0.86 for the self-discipline and impulse-control dimensions, respectively. The factor loadings for each item on the scale ranged from 0.81 to 0.88, and they had good construct validity [57]. In this study, the Cronbach’s alpha on the whole scale was 0.797, and the dimensions of self-discipline and impulse control were 0.803 and 0.746, respectively.

#### 2.2.3. Physical Activity

The International Physical Activity Questionnaire (IPAQ) was adopted to measure the individual PA, which has both short and long versions. In this study, we used the short version (IPAQ-SF) with seven items (specific items refer to Appendix A) to measure the PA level of the participants. The IPAQ-SF is a self-report scale that asked the participant about the time they spent being physically active in the last seven days. These physical activities were classified into three MET levels (metabolic equivalent means the amount of oxygen consumption required to maintain resting metabolism and represents the relative energy metabolism level), walking: 3.3 METs (e.g., walking for work and exercise leisure), moderate intensity: 4.0 METs (e.g., carrying light loads), and vigorous intensity: 8.0METs (e.g., lifting heavy loads). IPAQ scoring was expressed as MET-min per week: MET level *minutes of activity/day * days per week. Thus, total MET-minutes/week = 3.3 (METs * min * days) + 4.0 (METs * min * days) + 8.0 (METs * min * days) (Table 1). The reader can find the rules for classifying the three levels of MET in the Guidelines for Data Processing and Analysis of the International Physical Activity Questionnaire Guidelines [58]. The IPAQ is a culture-appropriate test questionnaire showing a promising PA measure among people [59]. The Chinese version of IPAQ is valid and reliable for assessing PA among Chinese people [60,61].

### 2.3. Data Analysis

This study used IBM^®^ SPSS^®^ Statistics 26.0 (IBM, Armonk, NY, USA) with a plug-in unit Process V3.5 and Amos 26.0 to manage and analyze the data. Process macro was a calculation program that used a regression-based process to test mediation, regulation, and conditional process analysis. Before analysis, if participants had incomplete data, more than 10% of the data were replaced by using Missing Value Analysis in SPSSS software [62]. We excluded outliers in total minutes of physical activity on the basis of IPAQ scoring guidelines [58]. Secondly, we used histograms and the Kolmogorov-Smirnoff (K-S test) for distributions of variables. We used two-tailed independent sample t-tests for normally distributed variables and Mann–Whitney’s U for non-normally distributed variables. Thirdly, we used Amos 26.0 for model fitting and path analysis. Finally, we investigated the moderating effect of gender differences on the indirect relationship between PA and self-efficacy.

We used Harman’s single-factor test to test the common method bias and analyzed all variables by non-rotating principal component analysis. The test results showed that there were four factors with eigenvalues greater than 1, and the contribution rate of the first factor was 34.05%, far lower than the critical value of 40% [63]. These results indicated that there was no serious common method deviation in this study.

## 3. Results

### 3.1. Preliminary Analysis

Descriptive statistics on college students’ self-efficacy, self-control, self-discipline, impulse control, and PA are presented in Table 2 by means and standard deviations (SD) or percentages(%).

Table 3 shows the bivariate correlations between the investigated variables. Self-efficacy was moderately and significantly related to PA (r = 0.16, *p* < 0.001). In addition, self-efficacy was positively and significantly related to self-discipline (r = 0.42, *p* < 0.001) and impulse control (r = 0.28, *p* < 0.001). Self-discipline (r = 0.23, *p* < 0.001) and impulse control (r = 0.12, *p* < 0.001) were positively and significantly related to PA.

### 3.2. Direct Effects and Mediation Results

Before the mediation analysis, we conducted variance inflation factors (VIFs) and tolerance tests to ensure no multicollinearity between independent variables in the proposed mediation model. For the intermediary model, all VIFs ranged from 1.05 to 1.20, and all tolerance scores ranged from 0.83 to 0.86, indicating no multicollinearity.

**Figure 1:**
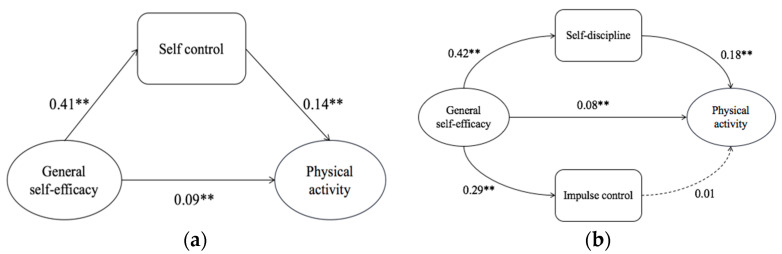
Mediating effect of self-control: (**a**) mediation model; (**b**) parallel mediation model (** *p* < 0.01).

We performed mediation analysis using Model 4 of Process macro of SPSS. To analyze the parallel multi-mediation model of the relationship between self-efficacy and PA, the bias-corrected bootstrap method was performed on the basis of 5000 bootstrap iterations and confidence intervals (CI) of 95%. Figure 1 shows the mediation model and parallel mediation model.

Table 4 shows the direct and indirect effects of self-efficacy on PA. The results of the parallel multiple mediation analysis indicated that the total effect of self-efficacy on PA was 0.15, SE = 0.05, *p* < 0.001, 95% CI [0.21, 0.40]. The direct effect of self-efficacy on PA acted as a significant predictor, with an effect of 0.08, SE = 0.05, *p* < 0.05, 95% CI [0.05, 0.26], explaining 49.93% of the total effect. The mediating effect of self-discipline on self-efficacy and PA was significant, with an effect of 0.07, SE = 0.01, 95% CI [0.05, 0.10], accounting for 48.69% of the total effect. The mediating effect of impulse control on self-efficacy and PA was insignificant, with an effect of 0.00, SE = 0.01, 95% CI [−0.01, 0.02], accounting for 1.38% of the total effect. The total indirect effect of self-efficacy on PA was 0.08, SE = 0.01, 95% CI [0.05, 0.10]. The results showed that self-discipline had a more significant mediating effect than impulse control.

### 3.3. Gender Difference

Considering the random sample and the large sample size, we conducted independent sample z-tests to examine mean-level differences of these variables regarding gender. As shown in Table 5, on average, males had higher mean level scores on self-discipline and PA than females. However, statistically, we found significant differences only in one variable, PA. We found no statistically significant differences between males and females in self-control, self-discipline, impulse control, and self-efficacy.

Using the multi-group analysis function of IBM^®^ SPSS^®^Amos 26.0, we examined the differences in path coefficients between male and female models and whether the entire model was equally valid across genders. As shown in Table 6, Model 1 (unconstrained model) has the restriction that all coefficients are allowed to vary across genders; Model 2 restricted the structural co-variances to be equal; and Model 3 restricted the structural residuals to be equal. The χ^2^ differences were insignificant, and the indicators all met the relevant requirements compared with other parameters.

**Figure 2:**
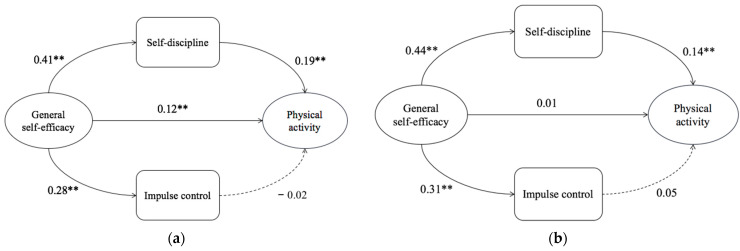
Parallel mediation model: (**a**) males; (**b**) females (** *p* < 0.01).

However, since χ^2^ was easily affected by the large sample size, to verify the cross-gender stability, the critical ratio of differences (CRD) was considered as an indicator to further investigate the span of the structural model. According to decision-making rules, the absolute value of CRD greater than 1.96 indicates a significant difference between the two parameters [64]. The results showed that CRD [self-efficacy → PA] = −2.103, which indicated that there was a significant difference between males and females on the path from self-efficacy to physical activity, while there was no significant difference between the variable structure paths (CRD [self-efficacy → self-discipline] = 0.865, CRD [self-efficacy → impulse control] = 0.809, CRD [self-discipline → PA] = −0.905, CRD [impulse control→ PA] = 1.222). Figure 2 depicts the structural model of male and female gender differences.

## 4. Discussion

The purpose of the present study was to examine the relationship between self-efficacy and PA through the potential mediation of self-discipline and impulse control as two subscales. The modified structural equation modeling results fit the indices. Moreover, the findings of the path analysis showed that in the parallel two-mediator model, only self-discipline mediated between general self-efficacy and physical activity, and impulse control did not play a mediating role. The results showed that self-efficacy has a significant direct effect on PA and self-discipline. Therefore, we concluded that self-control is a meaningful concept for understanding the mechanism of self-efficacy in PA. Specifically, the results revealed that a higher sense of self-efficacy predicted a higher sense of self-control and a higher level of PA. Consistent with the above hypothesis, self-efficacy could predict PA through continuous indirect effects. Comparing the two subscales, self-discipline could predict as opposed to than impulse control. Regarding the gender difference according to multi-group analysis, findings showed that the model path changed, in which self-efficacy is related directly to physical activity and indirectly via self-discipline for males. In contrast, for females, self-efficacy predicts PA only through self-discipline. There were no statistically significant differences in other paths between males and females.

### 4.1. Mediating Effect of Self-Control

The results showed that self-discipline, as a subscale of self-control, mediated the relationship between self-efficacy and PA in university students, and impulse control did not mediate the relationship, suggesting that self-discipline plays a more significant role than impulse control.

Previous studies have indicated that self-control strongly correlates with self-efficacy [65,66,67]. For example, in a study about procrastination and sleep problems among young adults, self-control positively affected self-efficacy [68]. Another study about low- and middle-income black children illustrated that better self-control in early childhood leads to better self-efficacy in adolescents [69]. Studies also confirmed the relationship between self-control and self-efficacy in other contexts, such as substance abuse [70,71], COVID-19 pandemic lockdown impact on psychological health [72], academic procrastination [73] and fitness performance [74]; it was also closely related to self-regulation and optimism [67], creative performance [75], behavior planning [21], law-related morality [76], and cognitive strategies [77].

Meanwhile, self-control was positive with physical activity too. According to the strength model of self-control, high self-control might help people to translate their PA intentions into action [78]. Kopp et al. (2020) showed that higher gym attendance was associated with improved self-control [79]. Camp et al. found significant differences in self-control between physically active and inactive UK adults after one year of COVID restrictions [72]. A meta-analysis demonstrated that acute aerobic exercise improves subsequent interference control performance [80]. Moreover, a study showed that students’ self-control was related to their exercise levels and that self-efficacy mediated the relationship between self-control and PA level during their final examination period [70]. All of these studies above confirmed the strong correlation between self-control and self-efficacy, or PA. To our knowledge, this study was likely the first to identify the mediating role of self-discipline between self-efficacy and PA. Perseverance to exercise constitutes a prime example of behavior that requires a person to exert self-control [42], and self-discipline failure could lead to physical inactivity. One study showed that self-discipline could independently predict exercise behavior [23]. As a motivational variable, it linked the TPB constructs to exercise behavior, and this result is consistent with our study. In the study, we highlight the critical mediating role of self-discipline, a dimension of self-control, in the relationship between self-efficacy and PA.

### 4.2. Gender Difference

The results indicate significant differences in the pathways of the mediation model for males and females, and the structural equation modeling supports this result. Self-efficacy in girls acts on PA to be achieved through the mediation of self-discipline, while males were going through two paths of self-efficacy directly to PA as well as partial mediating effect of self-discipline. The results imply that the self-discipline of females seems to be more important in improving their PA. In reality, females tend to have more psychological problems in their adolescence, such as depression and anxiety, which could represent obstacles during the pursuit of and adherence to goals [81,82,83]. In adolescent socialization, females are more interpersonally oriented and spend much more time on relationship-type activities than males [84]. In terms of physical activities during college, compared with females, males prefer to attend extracurricular activities. When males have more controllable time and better sports venues, they are more likely to carry out more physical activities during college. By taking part in physical activities, an individual could gain confidence and enthusiasm to focus more, which could further enhance one’s self-control through positive feedback, partially explaining the gender difference.

### 4.3. Limitations and Implications

There are some limitations to this research. Firstly, this research design was cross-sectional, so the result could not infer the causal relationship between variables. Secondly, one university in China provided the samples selected in this study, and future studies should broaden the representativeness of the samples to different groups and ages. Lastly, the data collection adopted a self-report method, and the data filling was vulnerable to individuals’ subjective responses. According to the above information, future studies need improvements to these limitations.

In addition to the above theoretical significance mentioned above, the study has some practical significance during the recurrent outbreak of COVID-19. The results indicated that self-control positively correlates with PA. Lacking PA would increase the risk of diseases, both physically and mentally. Therefore, educators and tutors should conduct mental health care to encourage students to improve self-control.

## 5. Conclusions

This study demonstrated the mediating role of self-control in self-discipline, not impulse control, between self-efficiency and PA. The model and related results expand our understanding of the relationship between self-efficacy and PA. The perspective of mediating mechanisms develops and specifies the theoretical views regarding how self-control leads to PA in self-discipline and impulse control. There was no difference between males and females in the level of self-control and its two dimensions, self-discipline and impulse control. However, males can promote PA levels by enhancing self-efficacy and self-discipline, whereas females require the full mediating effect of self-discipline. This study has practical significance for the ongoing global impact of COVID-19 restrictions on physical activity and mental health.

## Figures and Tables

**Table 1:** Physical activity statistical methods and results.

	Items	MET Level	Calculation Formula	Mean ± SD (MET-Min Per Week)
Walking	5, 6	3.3	3.3 * min * days	1029.68 ± 891.32
Moderate	3, 4	4.0	4.0 * min * days	1135.93 ± 945.07
Vigorous	1, 2	8.0	8.0 * min * days	1212.25 ± 1061.61

**Table 2:** Descriptive data for main variables.

	Total Sample (N = 1627)
Male, n (%)	19.41 (0.66)
Age in years, M (SD)	968 (59.6%)
Self-efficacy, M (SD)	2.51 (0.50)
Self-control, M (SD)	3.14 (0.86)
Self-discipline, M (SD)	3.21 (0.84)
Impulse control, M (SD)	3.09 (0.78)
PA (MET-min/week) PA, n (%)	3377.86 (2058.08)
Low	42 (2.6%)
Medium	726 (44.1%)
High	877 (53.3%)

**Table 3:** Correlation analysis.

	1	2	3	4	5
1. Self-efficacy	1				
2. Self-control	0.40 **	1			
3. Self-discipline	0.42 **	0.78 **	1		
4. Impulse control	0.28 **	0.87 **	0.40 **	1	
5. PA	0.16 **	0.20 **	0.23 **	0.12 **	1

** *p* < 0.01.

**Table 4:** Direct and indirect effects of self-efficacy on PA.

Outcome Variable	Predictive Variable	R	R-sq	F	Estimate	SE	*t*	*p*	95% CI	
									Lower	Upper
Self-discipline		0.42	0.17	338.59						
	Self-efficacy				0.42	0.04	18.67	0.000 ***	0.63	0.78
Impulse control		0.29	0.09	150.99						
	Self-efficacy				0.29	0.04	12.29	0.000 ***	0.38	0.52
PA		0.22	0.05	28.24						
	Self-efficacy				0.08	0.05	2.82	0.004 **	0.05	0.26
	Self-discipline				0.18	0.03	6.19	0.000 ***	0.14	0.28
	Impulse control				0.01	0.03	0.27	0.789	−0.06	0.08

** *p* < 0.01; *** *p* < 0.001.

**Table 5:** Independent sample z-test of gender difference.

	Gender	M ± SD	z	*p*
Self-control	Male	3.14 ± 0.65	−0.09	0.930
	Female	3.14 ± 0.73		
Self-discipline	Male	3.24 ± 0.82	−1.68	0.092
	Female	3.17 ± 0.86		
Impulse control	Male	3.06 ± 0.76	−1.45	0.147
	Female	3.12 ± 0.80		
Self-efficacy	Male	2.51 ± 0.51	−0.24	0.811
	Female	2.51 ± 0.48		
PA ^#^	Male	3492.30 ± 2112.65	−2.95	0.003 **
	Female	3209.75 ± 1964.71		

^#^: MET-min/week, M: mean; SD: standard deviation, ** *p* < 0.01.

**Table 6:** Multi-group analysis: males vs. females.

Model	Specifications	χ^2^	df	CFI	RMSEA	Model Comparison	χ^2^ Diff.	df Diff.	*p*
1	Unconstrained	2.604	1	0.998	0.031				
2	Structural co-variances	12.303	7	0.993	0.022	1 vs. 2	9.699	6	0.138
3	Structural residuals	12.676	9	0.995	0.016	1 vs. 3	10.072	8	0.260

## Data Availability

Data in the study are not publicly available to protect the participants’ privacy.

## Appendix A. Chinese IPAQ

1. During the last 7 days, on how many days did you do vigorous physical activities like heavy lifting, digging, aerobics, or fast bicycling?		
days per week	□ No vigorous physical activities→Skip to question 3	
2. How much time did you usually spend doing vigorous physical activities on one of those days?		
hours per day	minutes per day	□ Don’t know/Not sure
3. During the last 7 days, on how many days did you do moderate physical activities like carrying light loads, bicycling at a regular pace, or doubles tennis?		
days per week	□ No moderate physical activities→Skip to question 5	
4. How much time did you usually spend doing moderate physical activities on one of those days?		
hours per day	minutes per day	□ Don’t know/Not sure
5. During the last 7 days, on how many days did you walk for at least 10 minutes at a time?		
days per week	□ No Walking→Skip to question 7	
6. How much time did you usually spend walking on one of those days?		
hours per day	minutes per day	□ Don’t know/Not sure
7. During the last 7 days, how much time did you spend sitting on a week day?		
hours per day	minutes per day	□ Don’t know/Not sure
